# Bis[6-(3,5-dimethyl-1*H*-pyrazol-1-yl)picolinato-κ^2^ *N* ^1^,*O* ^2^]cadmium(II) 1.75-hydrate

**DOI:** 10.1107/S1600536808002444

**Published:** 2008-01-30

**Authors:** Zhao Kai, Xian-Hong Yin, Feng Yu, Jie Zhu, Cui-Wu Lin

**Affiliations:** a College of Chemistry and Ecological Engineering, Guangxi University for Nationalities, Nanning 530006, People’s Republic of China; b College of Chemistry and Chemical Engineering, Guangxi University, Nanning 530004, People’s Republic of China

## Abstract

In the title complex, [Cd(C_11_H_10_N_3_O_2_)_2_]·1.75H_2_O, the Cd atom is coordinated by four N atoms and two O atoms from two tridentate 6-(3,5-dimethyl-1*H*-pyrazol-1-yl)picolinate ligands in a distorted *cis*-N_4_O_2_ octa­hedral geometry. Three water mol­ecules, with occupancies of 1.0, 0.5 and 0.25, complete the asymmetric unit. The components of the crystal structure are linked *via* hydrogen bonds, forming a three-dimensional network.

## Related literature

For related literature, see: Zhao *et al.* (2007 ▶); Yin *et al.* (2007 ▶).
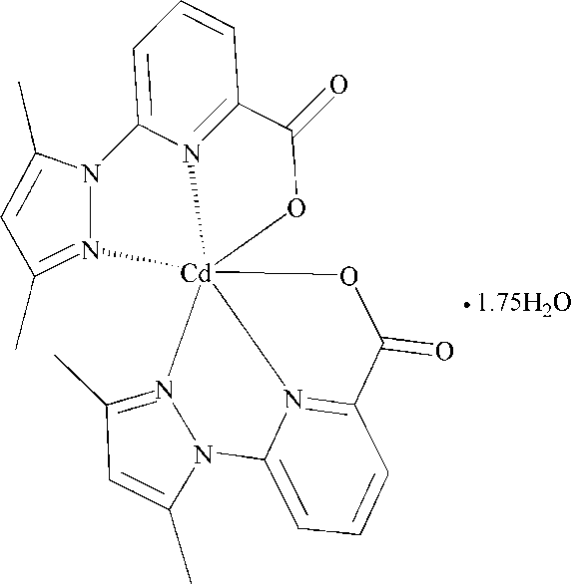


## Experimental

### 

#### Crystal data

[Cd(C_11_H_10_N_3_O_2_)_2_]·1.75H_2_O
*M* *_r_* = 576.38
Triclinic, 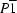

*a* = 9.7503 (9) Å
*b* = 11.4398 (15) Å
*c* = 12.843 (2) Å
α = 63.905 (1)°
β = 72.253 (1)°
γ = 82.688 (2)°
*V* = 1225.2 (3) Å^3^
*Z* = 2
Mo *K*α radiation
μ = 0.94 mm^−1^
*T* = 298 (2) K
0.52 × 0.48 × 0.43 mm

#### Data collection

Bruker SMART CCD area-detector diffractometer
Absorption correction: multi-scan (*SADABS*; Sheldrick, 1996 ▶) *T*_min_ = 0.641, *T*_max_ = 0.688
6152 measured reflections
4218 independent reflections
3279 reflections with *I* > 2σ(*I*)’
*R*_int_ = 0.024

#### Refinement

*R*[*F* ^2^ > 2σ(*F* ^2^)] = 0.041
*wR*(*F* ^2^) = 0.129
*S* = 1.02
4218 reflections
325 parameters
6 restraints
H-atom parameters constrained
Δρ_max_ = 0.86 e Å^−3^
Δρ_min_ = −0.68 e Å^−3^

### 

Data collection: *SMART* (Siemens, 1996 ▶); cell refinement: *SAINT* (Siemens, 1996 ▶); data reduction: *SAINT*; program(s) used to solve structure: *SHELXS97* (Sheldrick, 2008 ▶); program(s) used to refine structure: *SHELXL97* (Sheldrick, 2008 ▶); molecular graphics: *SHELXTL* (Sheldrick, 2008 ▶); software used to prepare material for publication: *SHELXTL*.

## Supplementary Material

Crystal structure: contains datablocks I, global. DOI: 10.1107/S1600536808002444/tk2242sup1.cif

Structure factors: contains datablocks I. DOI: 10.1107/S1600536808002444/tk2242Isup2.hkl

Additional supplementary materials:  crystallographic information; 3D view; checkCIF report

## Figures and Tables

**Table 1:** Hydrogen-bond geometry (Å, °)

*D*—H⋯*A*	*D*—H	H⋯*A*	*D*⋯*A*	*D*—H⋯*A*
O5—H5*A*⋯O2^i^	0.85	1.97	2.817 (7)	177
O5—H5*B*⋯O4^ii^	0.85	1.99	2.843 (7)	177
O6—H6*A*⋯O5	0.85	1.85	2.704 (15)	176
O6—H6*B*⋯O6^iii^	0.85	2.13	2.98 (4)	177
O7—H7*D*⋯O2^iv^	0.85	2.13	2.98 (3)	177
O7—H7*E*⋯O2^ii^	0.85	2.20	3.05 (3)	177

Symmetry codes: (i) 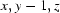; (ii) 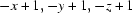; (iii) 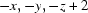; (iv) 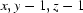.

**Fig. 1.**
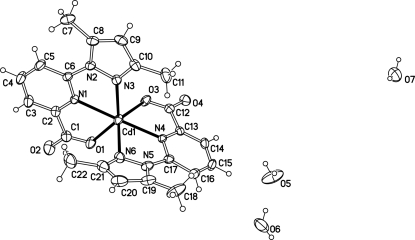
The molecular structure of (I) showing 50% probability displacement ellipsoids and the atom-numbering scheme.

**Fig. 2.**
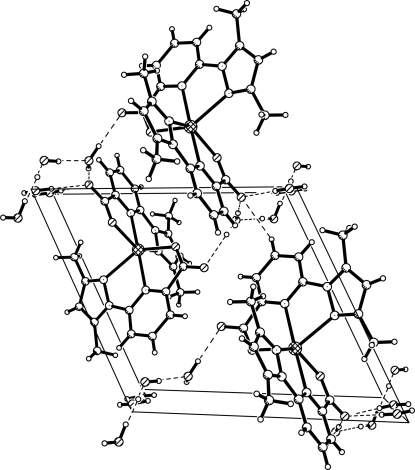
Crystal packing in (I) showing the hydrogen bonding interactions as dashed lines.

## supplementary crystallographic information

### Comment

Recently, we reported the crystal structures of bis(6-(3,5-dimethyl-1*H*-pyrazol-1-yl)picolinato)zinc(II) trihydrate (Yin *et al.*, 2007) and bis[3-chloro-6-(3,5-dimethyl-1*H*-pyrazol-1-yl)picolinato]cobalt(II) 2.5-hydrate (Zhao *et al.*, 2007). As a continuation of these investigations, we report the crystal structure of bis[6-(3,5-dimethyl-1*H*-pyrazol-1-yl)picolinato]cadmium(II) 1.75-hydrate, (I), herein, Fig. 1.

The asymmetric unit comprises a mononuclear cadmium(II) complex and three uncoordinated water molecules, with 100, 50, and 25% occupancy, respectively. The Cd atom is six-coordinated by four N atoms and two O atoms derived from the tridentate ligands. These define a distorted *cis*-N_4_O_2_ octahedral environment. The angles around the Cd(II) atom range from 68.77 (14) to 173.70 (13)°, the Cd—N distances range from 2.287 (4) to 2.383 (4) Å, and the Cd—O distances are 2.255 (4) to 2.276 (3) Å.

In the crystal structure, the ligand-O atoms and lattice water molecules participate in the formation of intermolecular hydrogen bonds that serve to link the components into a 3-D network, Fig. 2; for symmetry codes see Table 2.

### Experimental

6-(3,5-Dimethyl-1*H*-pyrazol-1-yl)picolinic acid (1 mmol, 217 mg) was dissolved in anhydrous ethyl alcohol (15 ml, AR, 99.9%) and stirred to give a clear solution. To this solution was added CdCl_2_.6H_2_O (0.5 mmol,149 mg) in anhydrous alcohol (10 ml). After evaporating the resulting solution in air to about half the volume, colorless blocks of (I) were formed. The crystals were isolated, washed with alcohol three times (Yield 75%). Analysis found: C 44.02, H 4.48, N 14.13; C_22_H_26_CdN_6_O_7_ requires: C 44.12, H 4.38, N 14.03.

### Refinement

The C-bound atoms were positoned geometrically and refined using a riding model with C—H = 0.93 - 0.96 Å, and with *U*_iso_(H) = 1.2*U*_eq_(C). The water-bound H atoms were located in difference Fourier maps and the O—H distances were constrained to 0.85 Å, with *U*_iso_(H) = 1.2*U*_eq_(O).

### Crystal data

**Table:**

[Cd(C_11_H_10_N_3_O_2_)_2_]·1.75H_2_O	*Z* = 2
*M**_r_* = 576.38	*F*_000_ = 583
Triclinic, *P*1	*D*_x_ = 1.562 Mg m^−^^3^
Hall symbol: -P 1	Mo *K*α radiation λ = 0.71073 Å
*a* = 9.7503 (9) Å	Cell parameters from 3283 reflections
*b* = 11.4398 (15) Å	θ = 2.6–27.4º
*c* = 12.843 (2) Å	µ = 0.94 mm^−^^1^
α = 63.905 (1)º	*T* = 298 (2) K
β = 72.253 (1)º	Block, colorless
γ = 82.688 (2)º	0.52 × 0.48 × 0.43 mm
*V* = 1225.2 (3) Å^3^	

### Data collection

**Table:**

Bruker SMART CCD area-detector diffractometer	4218 independent reflections
Radiation source: fine-focus sealed tube	3279 reflections with *I* > 2σ(*I*)'
Monochromator: graphite	*R*_int_ = 0.024
*T* = 298(2) K	θ_max_ = 25.0º
φ and ω scans	θ_min_ = 1.8º
Absorption correction: multi-scan(SADABS; Sheldrick, 1996)	*h* = −11→9
*T*_min_ = 0.641, *T*_max_ = 0.688	*k* = −12→13
6152 measured reflections	*l* = −15→14

### Refinement

**Table:**

Refinement on *F*^2^	Secondary atom site location: difference Fourier map
Least-squares matrix: full	Hydrogen site location: inferred from neighbouring sites
*R*[*F*^2^ > 2σ(*F*^2^)] = 0.041	H-atom parameters constrained
*wR*(*F*^2^) = 0.129	*w* = 1/[σ^2^(*F*_o_^2^) + (0.0751*P*)^2^ + 0.9467*P*] where *P* = (*F*_o_^2^ + 2*F*_c_^2^)/3
*S* = 1.02	(Δ/σ)_max_ = 0.001
4218 reflections	Δρ_max_ = 0.86 e Å^−^^3^
325 parameters	Δρ_min_ = −0.68 e Å^−^^3^
6 restraints	Extinction correction: none
Primary atom site location: structure-invariant direct methods	

### Special details

**Table:**

Geometry. All e.s.d.'s (except the e.s.d. in the dihedral angle between two l.s. planes) are estimated using the full covariance matrix. The cell e.s.d.'s are taken into account individually in the estimation of e.s.d.'s in distances, angles and torsion angles; correlations between e.s.d.'s in cell parameters are only used when they are defined by crystal symmetry. An approximate (isotropic) treatment of cell e.s.d.'s is used for estimating e.s.d.'s involving l.s. planes.
Refinement. Refinement of *F*^2^ against ALL reflections. The weighted *R*-factor *wR* and goodness of fit *S* are based on *F*^2^, conventional *R*-factors *R* are based on *F*, with *F* set to zero for negative *F*^2^. The threshold expression of *F*^2^ > σ(*F*^2^) is used only for calculating *R*-factors(gt) *etc*. and is not relevant to the choice of reflections for refinement. *R*-factors based on *F*^2^ are statistically about twice as large as those based on *F*, and *R*- factors based on ALL data will be even larger.

### Fractional atomic coordinates and isotropic or equivalent isotropic displacement parameters (Å^2^)

**Table:**

	*x*	*y*	*z*	*U*_iso_*/*U*_eq_	Occ. (<1)
Cd1	0.76574 (4)	0.71553 (3)	0.72484 (3)	0.04444 (17)	
N1	0.8787 (4)	0.8990 (4)	0.6894 (3)	0.0350 (8)	
N2	1.0975 (4)	0.8125 (4)	0.6253 (3)	0.0387 (9)	
N3	1.0204 (4)	0.7057 (4)	0.6513 (4)	0.0415 (9)	
N4	0.6745 (4)	0.5202 (3)	0.7667 (3)	0.0328 (8)	
N5	0.7146 (4)	0.4398 (4)	0.9541 (4)	0.0479 (10)	
N6	0.7398 (5)	0.5662 (4)	0.9302 (4)	0.0503 (11)	
O1	0.5961 (4)	0.8510 (4)	0.7729 (4)	0.0644 (11)	
O2	0.5533 (5)	1.0342 (4)	0.7955 (5)	0.0830 (14)	
O3	0.7078 (4)	0.7321 (3)	0.5601 (3)	0.0534 (9)	
O4	0.6161 (4)	0.6328 (4)	0.4818 (3)	0.0581 (10)	
O5	0.2952 (6)	0.1394 (7)	0.7369 (5)	0.135 (3)	
H5A	0.3713	0.1057	0.7569	0.161*	
H5B	0.3185	0.2082	0.6716	0.161*	
O6	0.0393 (19)	0.1318 (18)	0.8994 (15)	0.213 (11)	0.50
H6A	0.1200	0.1304	0.8500	0.256*	0.50
H6B	0.0199	0.0553	0.9551	0.256*	0.50
O7	0.305 (3)	0.001 (2)	0.011 (2)	0.132 (9)	0.25
H7D	0.3733	0.0111	−0.0519	0.158*	0.25
H7E	0.3411	−0.0089	0.0669	0.158*	0.25
C1	0.6338 (6)	0.9548 (6)	0.7655 (5)	0.0527 (14)	
C2	0.7944 (5)	0.9887 (5)	0.7156 (5)	0.0433 (12)	
C3	0.8505 (7)	1.1029 (5)	0.6986 (6)	0.0599 (15)	
H3	0.7911	1.1656	0.7170	0.072*	
C4	0.9967 (7)	1.1206 (6)	0.6535 (6)	0.0664 (17)	
H4	1.0369	1.1977	0.6390	0.080*	
C5	1.0845 (6)	1.0273 (5)	0.6296 (5)	0.0578 (15)	
H5	1.1840	1.0385	0.6018	0.069*	
C6	1.0219 (5)	0.9156 (5)	0.6476 (4)	0.0382 (11)	
C7	1.3609 (6)	0.8880 (6)	0.5427 (6)	0.0665 (17)	
H7A	1.4514	0.8543	0.5114	0.100*	
H7B	1.3637	0.8994	0.6117	0.100*	
H7C	1.3435	0.9703	0.4816	0.100*	
C8	1.2426 (5)	0.7943 (6)	0.5790 (5)	0.0473 (13)	
C9	1.2542 (6)	0.6767 (6)	0.5757 (5)	0.0540 (14)	
H9	1.3386	0.6380	0.5483	0.065*	
C10	1.1149 (6)	0.6247 (5)	0.6212 (5)	0.0487 (13)	
C11	1.0667 (7)	0.4955 (7)	0.6404 (7)	0.077 (2)	
H11A	0.9714	0.4759	0.6948	0.116*	
H11B	1.1316	0.4291	0.6743	0.116*	
H11C	1.0664	0.4989	0.5645	0.116*	
C12	0.6518 (5)	0.6369 (5)	0.5651 (4)	0.0416 (11)	
C13	0.6241 (5)	0.5148 (5)	0.6841 (4)	0.0372 (11)	
C14	0.5509 (6)	0.4074 (5)	0.7068 (5)	0.0517 (14)	
H14	0.5167	0.4039	0.6480	0.062*	
C15	0.5297 (6)	0.3044 (5)	0.8199 (6)	0.0588 (15)	
H15	0.4807	0.2304	0.8376	0.071*	
C16	0.5799 (6)	0.3101 (5)	0.9059 (5)	0.0567 (14)	
H16	0.5637	0.2421	0.9828	0.068*	
C17	0.6560 (5)	0.4210 (5)	0.8744 (4)	0.0417 (11)	
C18	0.7540 (7)	0.2101 (7)	1.0987 (6)	0.090 (3)	
H18A	0.8141	0.1719	1.1521	0.135*	
H18B	0.7898	0.1876	1.0315	0.135*	
H18C	0.6573	0.1777	1.1414	0.135*	
C19	0.7551 (6)	0.3540 (6)	1.0541 (5)	0.0607 (16)	
C20	0.8047 (7)	0.4268 (8)	1.0948 (5)	0.074 (2)	
H20	0.8393	0.3963	1.1621	0.089*	
C21	0.7937 (6)	0.5554 (7)	1.0161 (6)	0.0636 (16)	
C22	0.8333 (8)	0.6763 (8)	1.0196 (7)	0.087 (2)	
H22A	0.8017	0.7516	0.9598	0.130*	
H22B	0.9359	0.6804	1.0030	0.130*	
H22C	0.7876	0.6740	1.0981	0.130*	

### Atomic displacement parameters (Å^2^)

**Table:**

	*U*^11^	*U*^22^	*U*^33^	*U*^12^	*U*^13^	*U*^23^
Cd1	0.0337 (2)	0.0442 (2)	0.0605 (3)	−0.00267 (15)	−0.01602 (17)	−0.02399 (19)
N1	0.031 (2)	0.037 (2)	0.039 (2)	−0.0005 (16)	−0.0127 (17)	−0.0159 (17)
N2	0.024 (2)	0.049 (2)	0.044 (2)	0.0005 (17)	−0.0108 (17)	−0.0197 (19)
N3	0.029 (2)	0.050 (2)	0.054 (2)	0.0018 (18)	−0.0138 (18)	−0.028 (2)
N4	0.0270 (19)	0.033 (2)	0.036 (2)	−0.0021 (15)	−0.0059 (16)	−0.0145 (17)
N5	0.038 (2)	0.054 (3)	0.043 (2)	−0.0051 (19)	−0.0132 (19)	−0.010 (2)
N6	0.048 (3)	0.063 (3)	0.050 (3)	−0.002 (2)	−0.017 (2)	−0.029 (2)
O1	0.0317 (19)	0.062 (2)	0.108 (3)	−0.0022 (17)	−0.009 (2)	−0.049 (2)
O2	0.048 (3)	0.081 (3)	0.140 (4)	0.019 (2)	−0.020 (3)	−0.073 (3)
O3	0.057 (2)	0.052 (2)	0.051 (2)	−0.0168 (18)	−0.0257 (17)	−0.0096 (17)
O4	0.054 (2)	0.079 (3)	0.053 (2)	−0.001 (2)	−0.0213 (18)	−0.034 (2)
O5	0.068 (3)	0.151 (5)	0.117 (5)	−0.031 (3)	−0.041 (3)	0.022 (4)
O6	0.203 (18)	0.28 (2)	0.193 (16)	−0.130 (16)	0.094 (14)	−0.196 (17)
O7	0.164 (13)	0.124 (11)	0.112 (11)	−0.001 (9)	−0.022 (8)	−0.065 (9)
C1	0.036 (3)	0.059 (3)	0.072 (4)	0.010 (3)	−0.018 (3)	−0.036 (3)
C2	0.042 (3)	0.040 (3)	0.054 (3)	0.006 (2)	−0.021 (2)	−0.021 (2)
C3	0.061 (4)	0.051 (3)	0.079 (4)	0.001 (3)	−0.022 (3)	−0.036 (3)
C4	0.061 (4)	0.049 (3)	0.094 (5)	−0.014 (3)	−0.020 (3)	−0.032 (3)
C5	0.041 (3)	0.058 (3)	0.072 (4)	−0.016 (3)	−0.010 (3)	−0.026 (3)
C6	0.032 (3)	0.044 (3)	0.036 (3)	−0.006 (2)	−0.011 (2)	−0.013 (2)
C7	0.033 (3)	0.082 (4)	0.071 (4)	−0.006 (3)	−0.009 (3)	−0.022 (3)
C8	0.028 (3)	0.069 (4)	0.044 (3)	0.002 (2)	−0.011 (2)	−0.023 (3)
C9	0.033 (3)	0.081 (4)	0.052 (3)	0.014 (3)	−0.011 (2)	−0.035 (3)
C10	0.041 (3)	0.063 (3)	0.057 (3)	0.015 (2)	−0.022 (2)	−0.037 (3)
C11	0.061 (4)	0.078 (4)	0.123 (6)	0.018 (3)	−0.028 (4)	−0.072 (4)
C12	0.028 (2)	0.055 (3)	0.047 (3)	0.003 (2)	−0.013 (2)	−0.026 (2)
C13	0.025 (2)	0.049 (3)	0.046 (3)	0.0018 (19)	−0.008 (2)	−0.029 (2)
C14	0.044 (3)	0.057 (3)	0.071 (4)	−0.003 (2)	−0.013 (3)	−0.043 (3)
C15	0.056 (4)	0.041 (3)	0.080 (4)	−0.014 (3)	−0.006 (3)	−0.031 (3)
C16	0.052 (3)	0.046 (3)	0.061 (4)	−0.008 (3)	−0.007 (3)	−0.016 (3)
C17	0.027 (2)	0.048 (3)	0.047 (3)	−0.003 (2)	−0.004 (2)	−0.020 (2)
C18	0.059 (4)	0.079 (5)	0.085 (5)	−0.018 (3)	−0.033 (4)	0.020 (4)
C19	0.041 (3)	0.077 (4)	0.042 (3)	−0.008 (3)	−0.010 (2)	−0.004 (3)
C20	0.060 (4)	0.111 (6)	0.039 (3)	−0.008 (4)	−0.020 (3)	−0.015 (4)
C21	0.047 (3)	0.100 (5)	0.060 (4)	−0.003 (3)	−0.015 (3)	−0.047 (4)
C22	0.082 (5)	0.127 (6)	0.088 (5)	−0.010 (4)	−0.031 (4)	−0.069 (5)

### Geometric parameters (Å, °)

**Table:**

Cd1—O1	2.255 (4)	C4—H4	0.9300
Cd1—O3	2.276 (3)	C5—C6	1.382 (7)
Cd1—N4	2.287 (4)	C5—H5	0.9300
Cd1—N1	2.295 (4)	C7—C8	1.498 (8)
Cd1—N3	2.380 (4)	C7—H7A	0.9600
Cd1—N6	2.383 (4)	C7—H7B	0.9600
N1—C2	1.328 (6)	C7—H7C	0.9600
N1—C6	1.340 (6)	C8—C9	1.354 (8)
N2—N3	1.375 (5)	C9—C10	1.399 (8)
N2—C8	1.386 (6)	C9—H9	0.9300
N2—C6	1.401 (6)	C10—C11	1.500 (8)
N3—C10	1.317 (6)	C11—H11A	0.9600
N4—C17	1.324 (6)	C11—H11B	0.9600
N4—C13	1.325 (6)	C11—H11C	0.9600
N5—C19	1.367 (7)	C12—C13	1.524 (7)
N5—N6	1.378 (6)	C13—C14	1.373 (7)
N5—C17	1.416 (6)	C14—C15	1.384 (8)
N6—C21	1.313 (7)	C14—H14	0.9300
O1—C1	1.242 (7)	C15—C16	1.365 (8)
O2—C1	1.238 (6)	C15—H15	0.9300
O3—C12	1.248 (6)	C16—C17	1.388 (7)
O4—C12	1.242 (6)	C16—H16	0.9300
O5—H5A	0.8499	C18—C19	1.487 (9)
O5—H5B	0.8500	C18—H18A	0.9600
O6—H6A	0.8500	C18—H18B	0.9600
O6—H6B	0.8500	C18—H18C	0.9600
O7—H7D	0.8500	C19—C20	1.357 (9)
O7—H7E	0.8500	C20—C21	1.384 (9)
C1—C2	1.529 (7)	C20—H20	0.9300
C2—C3	1.383 (7)	C21—C22	1.505 (9)
C3—C4	1.369 (9)	C22—H22A	0.9600
C3—H3	0.9300	C22—H22B	0.9600
C4—C5	1.366 (8)	C22—H22C	0.9600
			
O1—Cd1—O3	97.39 (15)	H7A—C7—H7B	109.5
O1—Cd1—N4	113.34 (13)	C8—C7—H7C	109.5
O3—Cd1—N4	71.10 (12)	H7A—C7—H7C	109.5
O1—Cd1—N1	71.73 (13)	H7B—C7—H7C	109.5
O3—Cd1—N1	112.62 (12)	C9—C8—N2	106.6 (5)
N4—Cd1—N1	173.70 (13)	C9—C8—C7	128.0 (5)
O1—Cd1—N3	139.53 (13)	N2—C8—C7	125.4 (5)
O3—Cd1—N3	98.17 (14)	C8—C9—C10	106.8 (5)
N4—Cd1—N3	107.02 (13)	C8—C9—H9	126.6
N1—Cd1—N3	67.80 (13)	C10—C9—H9	126.6
O1—Cd1—N6	93.58 (16)	N3—C10—C9	110.6 (5)
O3—Cd1—N6	139.53 (14)	N3—C10—C11	120.3 (5)
N4—Cd1—N6	68.77 (14)	C9—C10—C11	129.0 (5)
N1—Cd1—N6	107.83 (14)	C10—C11—H11A	109.5
N3—Cd1—N6	98.25 (15)	C10—C11—H11B	109.5
C2—N1—C6	120.7 (4)	H11A—C11—H11B	109.5
C2—N1—Cd1	116.4 (3)	C10—C11—H11C	109.5
C6—N1—Cd1	122.9 (3)	H11A—C11—H11C	109.5
N3—N2—C8	109.5 (4)	H11B—C11—H11C	109.5
N3—N2—C6	118.2 (4)	O4—C12—O3	125.8 (5)
C8—N2—C6	132.2 (4)	O4—C12—C13	117.0 (4)
C10—N3—N2	106.4 (4)	O3—C12—C13	117.2 (4)
C10—N3—Cd1	137.1 (3)	N4—C13—C14	121.5 (5)
N2—N3—Cd1	116.4 (3)	N4—C13—C12	114.8 (4)
C17—N4—C13	120.6 (4)	C14—C13—C12	123.7 (4)
C17—N4—Cd1	121.7 (3)	C13—C14—C15	117.8 (5)
C13—N4—Cd1	117.2 (3)	C13—C14—H14	121.1
C19—N5—N6	110.7 (5)	C15—C14—H14	121.1
C19—N5—C17	132.0 (5)	C16—C15—C14	120.9 (5)
N6—N5—C17	117.3 (4)	C16—C15—H15	119.6
C21—N6—N5	104.6 (5)	C14—C15—H15	119.6
C21—N6—Cd1	135.8 (4)	C15—C16—C17	117.5 (5)
N5—N6—Cd1	113.6 (3)	C15—C16—H16	121.2
C1—O1—Cd1	119.3 (3)	C17—C16—H16	121.2
C12—O3—Cd1	119.4 (3)	N4—C17—C16	121.6 (5)
H5A—O5—H5B	108.3	N4—C17—N5	114.8 (4)
H6A—O6—H6B	108.5	C16—C17—N5	123.6 (5)
H7D—O7—H7E	108.3	C19—C18—H18A	109.5
O2—C1—O1	126.1 (5)	C19—C18—H18B	109.5
O2—C1—C2	116.1 (5)	H18A—C18—H18B	109.5
O1—C1—C2	117.7 (5)	C19—C18—H18C	109.5
N1—C2—C3	121.4 (5)	H18A—C18—H18C	109.5
N1—C2—C1	114.8 (4)	H18B—C18—H18C	109.5
C3—C2—C1	123.8 (5)	C20—C19—N5	106.4 (6)
C4—C3—C2	117.7 (5)	C20—C19—C18	128.8 (6)
C4—C3—H3	121.2	N5—C19—C18	124.6 (6)
C2—C3—H3	121.2	C19—C20—C21	106.2 (5)
C5—C4—C3	121.3 (5)	C19—C20—H20	126.9
C5—C4—H4	119.4	C21—C20—H20	126.9
C3—C4—H4	119.4	N6—C21—C20	112.1 (6)
C4—C5—C6	118.3 (5)	N6—C21—C22	119.5 (6)
C4—C5—H5	120.8	C20—C21—C22	128.5 (6)
C6—C5—H5	120.8	C21—C22—H22A	109.5
N1—C6—C5	120.6 (5)	C21—C22—H22B	109.5
N1—C6—N2	114.5 (4)	H22A—C22—H22B	109.5
C5—C6—N2	124.9 (4)	C21—C22—H22C	109.5
C8—C7—H7A	109.5	H22A—C22—H22C	109.5
C8—C7—H7B	109.5	H22B—C22—H22C	109.5
			
O1—Cd1—N1—C2	1.5 (3)	O1—C1—C2—N1	−2.6 (7)
O3—Cd1—N1—C2	92.0 (3)	O2—C1—C2—C3	−2.7 (8)
N4—Cd1—N1—C2	−142.8 (11)	O1—C1—C2—C3	177.6 (5)
N3—Cd1—N1—C2	−178.2 (4)	N1—C2—C3—C4	−0.1 (9)
N6—Cd1—N1—C2	−86.4 (4)	C1—C2—C3—C4	179.8 (6)
O1—Cd1—N1—C6	179.7 (4)	C2—C3—C4—C5	−1.9 (10)
O3—Cd1—N1—C6	−89.7 (4)	C3—C4—C5—C6	2.4 (10)
N4—Cd1—N1—C6	35.4 (13)	C2—N1—C6—C5	−0.8 (7)
N3—Cd1—N1—C6	0.1 (3)	Cd1—N1—C6—C5	−179.0 (4)
N6—Cd1—N1—C6	91.9 (4)	C2—N1—C6—N2	179.1 (4)
C8—N2—N3—C10	−0.4 (5)	Cd1—N1—C6—N2	1.0 (5)
C6—N2—N3—C10	179.2 (4)	C4—C5—C6—N1	−1.0 (8)
C8—N2—N3—Cd1	−177.5 (3)	C4—C5—C6—N2	179.0 (5)
C6—N2—N3—Cd1	2.1 (5)	N3—N2—C6—N1	−2.0 (6)
O1—Cd1—N3—C10	−177.5 (5)	C8—N2—C6—N1	177.5 (4)
O3—Cd1—N3—C10	−65.9 (5)	N3—N2—C6—C5	178.0 (5)
N4—Cd1—N3—C10	6.8 (5)	C8—N2—C6—C5	−2.6 (8)
N1—Cd1—N3—C10	−177.0 (5)	N3—N2—C8—C9	0.5 (5)
N6—Cd1—N3—C10	77.0 (5)	C6—N2—C8—C9	−179.0 (5)
O1—Cd1—N3—N2	−1.6 (4)	N3—N2—C8—C7	−178.5 (5)
O3—Cd1—N3—N2	110.0 (3)	C6—N2—C8—C7	2.0 (9)
N4—Cd1—N3—N2	−177.3 (3)	N2—C8—C9—C10	−0.4 (6)
N1—Cd1—N3—N2	−1.1 (3)	C7—C8—C9—C10	178.6 (5)
N6—Cd1—N3—N2	−107.1 (3)	N2—N3—C10—C9	0.1 (6)
O1—Cd1—N4—C17	−87.0 (4)	Cd1—N3—C10—C9	176.3 (4)
O3—Cd1—N4—C17	−177.0 (4)	N2—N3—C10—C11	178.7 (5)
N1—Cd1—N4—C17	55.9 (13)	Cd1—N3—C10—C11	−5.0 (8)
N3—Cd1—N4—C17	90.0 (4)	C8—C9—C10—N3	0.2 (6)
N6—Cd1—N4—C17	−2.5 (3)	C8—C9—C10—C11	−178.3 (6)
O1—Cd1—N4—C13	84.8 (3)	Cd1—O3—C12—O4	−179.5 (4)
O3—Cd1—N4—C13	−5.3 (3)	Cd1—O3—C12—C13	0.8 (6)
N1—Cd1—N4—C13	−132.4 (11)	C17—N4—C13—C14	0.2 (7)
N3—Cd1—N4—C13	−98.3 (3)	Cd1—N4—C13—C14	−171.7 (4)
N6—Cd1—N4—C13	169.3 (3)	C17—N4—C13—C12	179.2 (4)
C19—N5—N6—C21	−0.6 (6)	Cd1—N4—C13—C12	7.3 (5)
C17—N5—N6—C21	−179.8 (4)	O4—C12—C13—N4	174.7 (4)
C19—N5—N6—Cd1	156.6 (3)	O3—C12—C13—N4	−5.5 (6)
C17—N5—N6—Cd1	−22.6 (5)	O4—C12—C13—C14	−6.3 (7)
O1—Cd1—N6—C21	−86.0 (6)	O3—C12—C13—C14	173.5 (5)
O3—Cd1—N6—C21	168.3 (5)	N4—C13—C14—C15	0.7 (7)
N4—Cd1—N6—C21	160.4 (6)	C12—C13—C14—C15	−178.2 (5)
N1—Cd1—N6—C21	−14.0 (6)	C13—C14—C15—C16	0.1 (8)
N3—Cd1—N6—C21	55.2 (6)	C14—C15—C16—C17	−1.7 (9)
O1—Cd1—N6—N5	126.6 (3)	C13—N4—C17—C16	−2.0 (7)
O3—Cd1—N6—N5	20.8 (4)	Cd1—N4—C17—C16	169.5 (4)
N4—Cd1—N6—N5	12.9 (3)	C13—N4—C17—N5	−179.7 (4)
N1—Cd1—N6—N5	−161.5 (3)	Cd1—N4—C17—N5	−8.2 (5)
N3—Cd1—N6—N5	−92.2 (3)	C15—C16—C17—N4	2.7 (8)
O3—Cd1—O1—C1	−114.5 (5)	C15—C16—C17—N5	−179.7 (5)
N4—Cd1—O1—C1	172.9 (4)	C19—N5—C17—N4	−158.2 (5)
N1—Cd1—O1—C1	−3.1 (4)	N6—N5—C17—N4	20.8 (6)
N3—Cd1—O1—C1	−2.6 (6)	C19—N5—C17—C16	24.1 (8)
N6—Cd1—O1—C1	104.5 (5)	N6—N5—C17—C16	−156.9 (5)
O1—Cd1—O3—C12	−110.0 (4)	N6—N5—C19—C20	0.8 (6)
N4—Cd1—O3—C12	2.2 (4)	C17—N5—C19—C20	179.8 (5)
N1—Cd1—O3—C12	176.8 (4)	N6—N5—C19—C18	−174.6 (5)
N3—Cd1—O3—C12	107.5 (4)	C17—N5—C19—C18	4.4 (9)
N6—Cd1—O3—C12	−5.6 (5)	N5—C19—C20—C21	−0.6 (7)
Cd1—O1—C1—O2	−175.6 (5)	C18—C19—C20—C21	174.5 (6)
Cd1—O1—C1—C2	4.1 (7)	N5—N6—C21—C20	0.2 (6)
C6—N1—C2—C3	1.4 (7)	Cd1—N6—C21—C20	−149.2 (5)
Cd1—N1—C2—C3	179.7 (4)	N5—N6—C21—C22	−179.3 (5)
C6—N1—C2—C1	−178.4 (4)	Cd1—N6—C21—C22	31.3 (9)
Cd1—N1—C2—C1	−0.1 (5)	C19—C20—C21—N6	0.3 (7)
O2—C1—C2—N1	177.1 (5)	C19—C20—C21—C22	179.7 (6)

### Hydrogen-bond geometry (Å, °)

**Table:**

*D*—H···*A*	*D*—H	H···*A*	*D*···*A*	*D*—H···*A*
O5—H5A···O2^i^	0.85	1.97	2.817 (7)	177
O5—H5B···O4^ii^	0.85	1.99	2.843 (7)	177
O6—H6A···O5	0.85	1.85	2.704 (15)	176
O6—H6B···O6^iii^	0.85	2.13	2.98 (4)	177
O7—H7D···O2^iv^	0.85	2.13	2.98 (3)	177
O7—H7E···O2^ii^	0.85	2.20	3.05 (3)	177

Symmetry codes: (i) *x*, *y*−1, *z*; (ii) −*x*+1, −*y*+1, −*z*+1; (iii) −*x*, −*y*, −*z*+2; (iv) *x*, *y*−1, *z*−1.

## References

[bb1] Sheldrick, G. M. (1996). *SADABS* University of Göttingen, Germany.

[bb2] Sheldrick, G. M. (2008). *Acta Cryst.* A**64**, 112–122. 10.1107/S0108767307043930 18156677

[bb3] Siemens (1996). *SMART* and *SAINT* Siemens Analytical X-ray Systems, Inc., Madison, Wisconsin, USA.

[bb4] Yin, X.-H., Zhao, K., Feng, Y. & Zhu, J. (2007). *Acta Cryst.* E**63**, m2926.

[bb5] Zhao, K., Yin, X.-H., Feng, Y. & Zhu, J. (2007). *Acta Cryst.* E**63**, m3024.

